# Supporting Trustworthy AI Through Machine Unlearning

**DOI:** 10.1007/s11948-024-00500-5

**Published:** 2024-09-11

**Authors:** Emmie Hine, Claudio Novelli, Mariarosaria Taddeo, Luciano Floridi

**Affiliations:** 1 Department of Legal Studies, University of Bologna, Via Zamboni, 27/29, 40121 Bologna, Italy; 2 Centre for IT & IP Law, KU Leuven, Sint-Michielsstraat 6, 3000 Leuven, Flanders, Belgium; 3 Oxford Internet Institute, University of Oxford, 1. St. Giles’, Oxford, OX1 3JS UK; 4 Digital Ethics Center, Yale University, 85 Trumbull St., New Haven, CT 06511 USA; 5 The Alan Turing Institute, British Library, 96 Euston Rd, London, NW1 2DB UK

**Keywords:** Machine unlearning, Machine learning, Trustworthy AI, GELSI, Technology policy

## Abstract

Machine unlearning (MU) is often analyzed in terms of how it can facilitate the “right to be forgotten.” In this commentary, we show that MU can support the OECD’s five principles for trustworthy AI, which are influencing AI development and regulation worldwide. This makes it a promising tool to translate AI principles into practice. We also argue that the implementation of MU is not without ethical risks. To address these concerns and amplify the positive impact of MU, we offer policy recommendations across six categories to encourage the research and uptake of this potentially highly influential new technology.

## Introduction

Machine unlearning (MU) is not, as its name may suggest, the inverse of machine learning (ML), although they are related. In ML, an algorithm trains a model to perform a task using some data (Mitchell, 2013, p. 1–14; Singh et al., 2016). MU does not involve “forgetting” a task, but how specific data contribute to a model (Bourtoule et al., 2020). In other words, it seeks to “undo” the influence of some data on an ML model. The data in question can be specific datapoints, classes, features (Nguyen et al., 2022a, 2022b), and labels (Warnecke et al., 2022). MU techniques are part of the broader field of “model disgorgement” (Achille et al., 2023) and are divided into two categories: exact unlearning, which involves some degree of retraining, and approximate unlearning, which does not retrain the model but alters its weights and/or architecture to resemble a model that had not learned from the data in question (Nguyen et al., 2022a, 2022b; Qu et al., 2023; Thudi et al., 2022). Some MU methods are model-type-agnostic; others target specific types of ML models (Nguyen et al., 2022a, 2022b). All MU techniques seek to be more efficient than the bluntest form of exact unlearning—retraining the model from scratch—while achieving a similar level of verifiable accuracy (Guo et al., 2020). In this journal in 2020, Morley et al. discussed the need for methods to translate ethical principles into practice (2020). This commentary posits that MU can be one of these methods, specifically to implement the principles of trustworthy AI. MU is a relatively new subfield, but its potential usefulness in mitigating AI risks is acknowledged in academic literature and industry. For example, Google announced its “first Machine Unlearning Challenge” to develop new machine unlearning techniques, specifically to address AI bias and privacy risks (Pedregosa & Triantafillou, 2023).

In this commentary, we argue that MU can be used to foster trustworthy AI and offer some recommendations to this end. We use the May 2024 update to the Organisation for Economic Co-operation and Development’s (OECD) five principles for innovative and trustworthy AI (OECD, 2024) as a benchmark because they provide a shared standard. Over the past years, many organizations have published ethical principles to foster trustworthy AI. The terminology varies, but there is significant overlap among these principles, as shown by Floridi and Cowls (2019) and Jobin et al. (2019). The OECD principles align with the identified commonalities and have been adopted by 42 countries (OECD, 2019). They also inspired the G20 principles, which have been adopted by yet more countries (“G20 Ministerial Statement on Trade & Digital Economy” 2019). Thus far, considerations of the governance, ethical, legal, and social implications (GELSI) of MU in the literature have tended to focus on the “right to be forgotten” in the European Union (EU) context (Floridi, 2023), which aims to ensure that EU citizens can have their personal information delisted by search engines or deleted by data controllers in some circumstances (Floridi, 2015). Less attention has been paid to other GELSI of MU for other ethical AI challenges (Taddeo & Floridi, 2018). We shall address this research gap, showing that MU has positive GELSI that extend beyond the right to be forgotten, while also considering the relevant ethical risks that must be considered so that MU is itself trustworthy. We argue that, with an adequate policy approach, risks could be mitigated while leveraging the positive GELSI, and offer policy recommendations to this end.

## The GELSI of Machine Unlearning for Trustworthy AI

Each of the five OECD principles encapsulates specific normative goals. In this section, we outline how MU could support them.

### Inclusive Growth, Sustainable Development, and Well-Being

The first OECD principle contains three normative goals: “inclusive growth, sustainable development and well-being” (OECD, 2024). MU can foster inclusive growth by empowering individuals with control over their data. This extends beyond the right to be forgotten, as it can also facilitate control of how ML processes use their data. However, inclusivity in a data-driven economy requires those with limited technological knowledge to be able to participate and exert control over their data in a meaningful way. Thus, MU methods need to be transparent and explainable so that people can understand their purposes and limitations, and the process of requesting unlearning must be easily accessible. This requires the unlearning request procedure to also be accessible. For example, while requesting it may be as easy as submitting an online form, the form itself should not obscured within a labyrinth of web pages requiring many clicks to access or an exorbitant fee, and the user should be informed that the unlearning has worked in a not overly technical way. We shall elaborate on the transparency and explainability requirements when discussing the normative goals of the third OECD principle.

Regarding the sustainable development goal,^1^ MU could reduce environmental impact by lessening the energy intensiveness of ML (García-Martín et al., 2019), including LLMs (Bender et al., 2021). For instance, training a single transformer with 213 million parameters is estimated to emit over 600,000 pounds of CO_2_, or the lifetime emissions of about five cars (Hao, 2019; Strubell et al., 2019); GPT-4, one of the largest transformer-based large language models (LLMs), is reported to have over 1 trillion parameters (Albergotti, 2023). AI and its data centers are also responsible for enormous freshwater consumption, estimated to reach 4.2–6.6 billion cubic meters in 2027 (Li et al., 2023). The resource demands of ML are growing rapidly as models become larger and their use spreads, with development and training being the most energy-intensive processes (Kaack et al., 2022). At the same time, demands that data be removed from models are growing through the right to be forgotten and, potentially, copyright lawsuits. However, merely deleting data from a training set is insufficient because various methods can still deduce the original training data or uncover information associated with the removed data, which persists in the model’s parameters. For instance, data from the training dataset can “leak” into the outputs of some ML models (De Cristofaro, 2020). This can happen in classification when a model is overfitted and a user queries a model with data identical, or very similar, to data in its training set, resulting in outputs that reflect the training inputs (Yeom et al., 2018). Generative models, such as LLMs and image generators, can also leak data by outputting memorized information (Floridi, 2023; Kodge et al., 2023), including personal identifiable information like email addresses and phone numbers (Nasr et al., 2023). The regurgitation of memorized copyrighted data is the foundation of the New York Times’s lawsuit against Microsoft and OpenAI (The New York Times Company v. Microsoft Corporation, 2023). Furthermore, training data can be vulnerable to exposure by attacks from specially trained generative adversarial networks (GANs) (Hitaj et al., 2017) or other membership inference attacks (Shokri et al., 2017).

To disgorge the influence of specific data, either full retraining or MU is necessary (Nguyen et al., 2022a, 2022b). In aggregate, retraining ML models every time a dataset is updated would be highly damaging to the environment.^2^ MU can lessen the need for full retraining, thus improving sustainability and making the investment in AI less of a gambit (Cowls et al., 2023). However, although MU’s goal is to be more efficient than full retraining (Cao & Yang, 2015), it must also be acknowledged that some MU methods are computationally intensive or require large neural networks, which could shut out small-scale providers and negate some of the energy savings of MU (Shaik et al., 2023). Thus, leveraging MU to achieve this goal requires adequate policy measures, which will be discussed in the recommendations, and a clear understanding of the trade-offs.

The third goal of the first principle is well-being, which largely stems from achieving the first two in a balanced way. However, although there are many aspects to well-being, MU can improve specific groups’ well-being by giving them more control over their data and enhancing ML model outputs. For instance, artists and authors are suing OpenAI and two companies offering AI art tools, alleging that the use of their works in training generative AI models violated their copyright by generating, for example, summaries and pieces of copyrighted material and images in the style of specific artists (Blistein, 2023; Vincent, 2023). In December of 2022, Stability.AI, which created the AI art generator Stable Diffusion, announced that they would give artists “around a couple of weeks” to opt out of using their works before training the next version of Stable Diffusion (Heikkilä, 2022). In this context, MU could give companies and artists more flexible options, with the ability to have their works removed from the model at any time in the future, after training is complete. MU could also reduce objectionable outputs by reshaping training datasets to be less biased or toxic, supporting the well-being of users and those harmed by model bias. However, these opportunities should be regulated to limit the risk of AI providers being given *carte blanche* to use any data in training with the justification that data may be removed later on.

### Respect for the Rule of Law, Human Rights and Democratic Values, Including Fairness and Privacy

The second OECD principle focuses on upholding the rule of law, human rights, and “democratic and human-centered values,” which include “non-discrimination and equality, freedom, dignity, autonomy of individuals, privacy and data protection, diversity, fairness, social justice, and internationally recognised labour rights” (OECD, 2024). Legally, the right to be forgotten (originally applied only to search engines but then formalized as the “right to erasure” in the General Data Privacy Regulation (GDPR) (Article 17(2)) upholds many of these normative goals (European Parliament and Council of the European Union, 2016). It enhances the right to privacy and the freedom not to be beholden to outdated or erroneous personal data, thereby safeguarding individual dignity and autonomy. However, the GDPR only states that the data must be “erased,” which is inadequate, when not inapplicable, for ML models that have trained on sources containing personal data, such as LLMs, because of the aforementioned possibility of models revealing their training data.

Currently, many LLMs attempt to “filter” requests that could violate terms of service. However, filtering does not remove data; it only tries to make it inaccessible and is not wholly effective. Even without access to the underlying model, simple “jailbreaks” can get around these filters and even cause models to reveal personally identifiable information present in their training data (Nasr et al., 2023). While filtering works (at least regionally) for search engines, which are given a predetermined set of links to delist, ML models remain vulnerable to attacks and exploitation that can bypass filters. Not only is MU a more efficient strategy to enforce the right to be forgotten, but it is also potentially necessary for its full realization. Although there is no case law yet on whether the right to be forgotten requires data to be expunged from models via MU, the spirit of the right suggests that it should. Indeed, the UK Information Commissioner’s Office issued guidance indicating that, in some cases, retraining the model or deleting it altogether would be necessary to facilitate the right (Information Commissioner’s Office, 2020). MU could be an effective alternative to full retraining or deletion.

MU is also valuable on a collective level, an aspect often overlooked in literature. Regarding the right to be forgotten, unlearning specific datapoints can support group privacy (Floridi, 2017), which could be especially important for marginalized groups and in contexts where group profiling is increasingly common. However, when discussing the limits of MU, we shall see it is crucial to ensure that the process does not compromise the accuracy of models or increase bias, as unlearning can affect classification model accuracy (Qu et al., 2023) and hence negatively impact the groups it is intended to benefit.

In line with the goals of the second OECD principle, MU can promote non-discrimination, equality, and fairness by unlearning not just specific datapoints, but also classes, labels, and features. Classes are categories of classification (for example, “cat” and “dog” in an image classifier sorting cats and dogs). Labels are categories assigned during training; they often overlap with classes. Features are a measurable property or characteristic of data used by a model to make predictions (for instance, snout length).^3^ Unlearning one or multiple classes (Poppi et al., 2023) can make models fairer when the problem definition has changed—perhaps an image classifier is adding classes that reflect more cultural nuance, but in the process needs to unlearn old classes—and correct biased labels. The same holds true of unlearning features (Warnecke et al., 2022). For example, eliminating gender or race features from a biased loan approval algorithm could make it fairer, although proxies for these features may remain. Therefore, it may also be necessary to remove discriminatory predictive features, such as postal codes, which could enhance fairness in a recidivism risk assessment algorithm (van Dijck, 2022). However, note that unlearning a feature is a radical solution because that feature cannot be used for any classification going forward, even if not one of the biased or unfair classifications. One study found that unlearning features can impact a large proportion of datapoints—up to 40% in one experiment (Warnecke et al., 2022). Thus, it may be desirable to unlearn specific datapoints or enhance the training data with additional datapoints, if possible, to achieve the same goals.

### Transparency and Explainability

MU can also support transparency and explainability, the third OECD principle (OECD, 2024). Even in cases where the model itself is a black box, MU can facilitate high-level transparency by giving individuals input into the machine learning process. Interestingly, MU could potentially bolster explainable AI by uncovering implicit relationships between ML models’ internal filters and the classes they contribute to, providing insight into black box models (Poppi et al., 2023). While nascent, this should be researched further to explore how MU could facilitate explainability more broadly.

As mentioned above, MU processes must themselves be transparent and explainable so that individuals can understand how MU can help them exert control over their data by understanding the “capabilities and limitations” of the model (OECD, 2024). To this end, in our Recommendations, we encourage that MU be “certifiable”—meaning that the model should be guaranteed to be within a specific threshold of performance as a model trained without the unlearned data (Guo et al., 2020)—so that affected entities know that unlearning has worked, and it must be explainable in “plain and easy-to-understand” terms (OECD, 2024), so that one can know why it worked.

### Robustness, Security, and Safety

The whole purpose of MU is to remove the influence of data from a model (Achille et al., 2023), which would also mitigate attacks and exploitation designed to access training data. This is why MU can support AI robustness by defending against attacks, making ML models less vulnerable. For example, MU can limit the impact of some data poisoning attacks, where data labels are flipped, incorrectly labeled data is introduced, or patterns are embedded in training data to create a “backdoor” and manipulate classifications (Gu et al., 2019; Tolpegin et al., 2020). One experiment showed that MU can correct maliciously altered labels, achieving near-baseline levels of accuracy in significantly less time than retraining the model (Warnecke et al., 2022). However, many “corrective machine unlearning” techniques rely on being able to identify manipulated data, although some techniques are being developed that can remove the influence of manipulated data based on a small subset (Goel et al., 2024).

As mentioned above, exploits, including membership inference attacks—which can determine, with high levels of accuracy, whether specific datapoints were used to train an ML model (Carlini et al., 2022; Shokri et al., 2017)—are among the major privacy risks to ML models. Limiting their impact is crucial to preserving privacy and upholding the right to be forgotten.

However, like other AI-related processes, MU must be secure and traceable to ensure “information integrity” (OECD, 2024) avoid attacks and misuse.^4^ MU algorithms could be vulnerable to attacks that increase computation costs (Marchant et al., 2022) or use malicious unlearning requests to skew the model—for example, if a model is made to unlearn a label introduced for debiasing efforts, or a set of datapoints that would, if deleted, cause the underrepresentation of a specific group in the training dataset. There is also evidence that MU models can make ML models vulnerable to novel membership inference attacks (Chen et al., 2021), and while MU can help counter some data poisoning attacks, it has been shown to create vulnerabilities through novel “camouflaged data poisoning attacks” (Di et al., 2022). Thus, MU processes should be carefully overseen to prevent tampering, but monitored carefully, certifiable MU techniques could be part of a certification scheme for trustworthy AI, or at least assist in self-regulation.

### Accountability

The final OECD principle concerns accountability for system functionality and for respecting the other principles (OECD, 2024). The OECD defines accountability as both compliance with AI system design, development, and deployment rules (proactive accountability) and demonstration of this compliance when failures occur (reactive accountability) (Novelli et al., 2023). In this context, MU techniques can support both proactive, ex-ante compliance with data quality and fairness standards by mitigating risks to privacy and ensuring the AI system’s proper operation throughout its lifecycle. This approach helps prevent unintended outcomes. It can also support reactive, ex-post measures through enhanced transparency, oversight, and explanations of undesired or biased results, as well as by enabling the right to be forgotten. Consequently, MU empowers affected parties to seek redress and enhances access to justice. However, companies need to incorporate proactive compliance with copyright and other laws and not use MU as a tool to escape accountability over data that should not have been used in the first place, such as child abuse images in the LAION-5B dataset used to train some image generators (David, 2023).

## Recommendations

In the last section, we discussed how MU may support trustworthy AI and instances in which special consideration is needed to ensure that it upholds the same principles. Any procedure that deletes or reverses a process or some of its parts may cause unwanted problems or be abused for unethical and illegal purposes if the manipulated element was ethically or legally required, or at least desirable. Thus, MU could be misused to undo what an ML process in line with the OECD principles has achieved and, for example, increase disinformation, bias, or unfairness. This is why it must be handled with ethical and legal supervision. At the same time, MU has great potential to facilitate trustworthy AI. To mitigate its risks and maximize its benefits, we offer the following recommendations derived from our analysis, beginning with issues that directly interface with users, moving into more technical requirements, and concluding with recommendations for the legal landscape of MU.

1. *Accessibility* To support inclusion and well-being, how MU processes are incorporated into platforms should be straightforward and user-friendly, enabling individuals of varying technical familiarities to understand how they function and easily request the removal of their data from machine learning models. To achieve this, a mix of technical measures and policy strategies is necessary. These may include creating intuitive user interfaces, standardizing MU protocols, and setting up dedicated channels within companies for addressing user requests and appeals.
2. *Transparency, explainability, and recordkeeping* This involves informing users or deployers of MU of the expected impacts on the model’s performance and its adherence to ethical and legal norms, before the process begins, through clear and reliable explanations at multiple levels of complexity. Decisions to rely on MU should rest on an assessment of the foreseeable impact of MU on model accuracy, bias, and other ethical and legal metrics. To support sustainability, MU energy use should also be calculated and weighed against the energy costs of retraining. Finally, dataset versioning, which consistently monitors and tracks alterations in the dataset, should be used to preserve the history of the varied dataset iterations used in model training, but kept secure to prevent malicious access.
3. *Accuracy* For MU to support trustworthy AI, safeguards should be in place to ensure that MU techniques do not (no matter how inadvertently) increase bias and unfairness or decrease accuracy by an unacceptable amount. To this end, impact assessments—e.g., of data protection and/or fundamental rights—should be performed (and documented) to understand potential shifts in accuracy and bias. Moreover, clear accuracy benchmarks—such as the “completeness” of unlearning, or how similar it is to the original model before unlearning (Cao & Yang, 2015), or a reasonable tolerance margin—should be set on a case-by-case basis to guide possible adjustments to the unlearning strategy if the performance of the model falls below these standards.
4. *Certifiability* For transparency purposes, unlearning must be “certifiable” so that affected entities know that the unlearning was completed and that the resulting model will closely resemble a model never trained on the unlearned data (Guo et al., 2020). A certifiable MU framework should provide users, regulators, and auditors with tools and tests to verify the success of the unlearning process (Nguyen et al., 2022a, 2022b).
5. *Cybersecurity* MU algorithms need consistent monitoring to prevent misuse through adversarial attacks and ensure robustness. Strategies include minimizing the data available to potential attackers or reducing the influence of individual data points on the model outcomes, a practice seen in differential privacy (Chen et al., 2021). Furthermore, systems should be implemented to identify irregular patterns or discrepancies in data that might signal data poisoning, such as real-time monitoring (Taddeo et al., 2019).
6. *Enforceability* Wherever local legislation allows it (e.g., in the EU), regulators should incentivize MU.^5^ This could be done by considering it as a (privacy) standard for conformity assessments; by including it within a comprehensive certification system for trustworthy AI; or by encouraging it in self-regulation, such as through incorporation into the “Assessment List for Trustworthy AI” (European Commission, 2020). In jurisdictions with less legislative action, like the US, MU could be incorporated into executive and voluntary governance measures. In the US, it could help enforce the Executive Order on the Safe, Secure, and Trustworthy Development and Use of Artificial Intelligence (Executive Office of the President, 2023), especially its provisions on mitigating the privacy risks of AI (Sec. 2(f)).

## Conclusion

MU is a novel subfield of ML that holds great promise as a technical measure to support trustworthy AI. We have argued that unlearning datapoints, features, labels, and classes can help translate ethical principles into practice (Morley et al., 2020) by helping AI applications uphold the OECD’s principles of trustworthy AI and ensuring that AI is more sustainable, inclusive, transparent, robust, and accountable. However, it is important to stress that MU cannot compensate for misuses of ML and the lack, or poor quality, of training data. For instance, after Google Photos was criticized in 2015 for classifying two Black people as “gorillas,” Google removed the “gorilla” category from search; Apple followed suit (Grant & Hill, 2023). As with any form of filtering, this did not solve the underlying problem of insufficient training data, specifically an underrepresentation of Black people (Grant & Hill, 2023). In this case, the solution would be to improve the quantity and quality of training data rather than attempting to identify the features that led to misclassifications and unlearn them. MU cannot compensate for insufficient training data, but it will be a crucial arrow in the quiver of tools promoting trustworthy AI.

Incorporating MU into existing processes and workflows, as well as in the legislative framework concerning AI, will not be a simple undertaking, but further research will help practitioners decide what specific techniques and algorithms to use. MU can support AI that is good for people, our communities, and our planet. Researchers, practitioners, and policymakers should invest in its development and application.

## Data Availability

Not applicable.

## Footnotes

1 While “sustainable development” covers more than just the environment and includes social and economic aspects, social well-being is covered elsewhere in this Commentary, and a full economic impact analysis is beyond the scope of this piece. Therefore, we focus in this section on environmental sustainability.

2 Additionally, retraining large models from scratch often results in in a model so different from the original model as to be unusable in a given workflow (Achille et al., 2023), requiring additional resources to develop a new model. Furthermore, the cost of training runs—in the millions of dollars for some LLMs (Meyer 2024)—means that retraining would be financially impractical for larger models.

3 In some cases, such as unlearning memorized sequences in an LLM, both features and labels must be unlearned (Warnecke et al., 2022).

4 This overlaps with part of the fifth principle, Accountability, on ensuring traceability, but as it relates to information integrity and security, we have elected to include it here.

5 While we believe that MU is a potent tool to achieve trustworthy AI, we avoid recommending that its use specifically be enshrined into law, as regulating technologies (as opposed to their impacts) can lead to laws that rapidly become outdated (Floridi 2020).
